# A preliminary examination of the effects of childhood abuse and resilience on pain and physical functioning in patients with knee osteoarthritis

**DOI:** 10.1515/sjpain-2023-0122

**Published:** 2024-06-06

**Authors:** JiHee Yoon, Ayeong (Jenny) Kim, Jenna M. Wilson, Jolin B. Yamin, Kristin L. Schreiber, Robert R. Edwards, Marise C. Cornelius, Claudia M. Campbell, Michael T. Smith, Jennifer A. Haythornthwaite, Christine B. Sieberg, Samantha M. Meints

**Affiliations:** Department of Anesthesiology, Perioperative, and Pain Medicine, Mass General Brigham, Harvard Medical School, 850 Boylston Street, Suite 308H, Chestnut Hill, Boston, MA 02467, United States of America; Center for Health Outcomes and Interdisciplinary Research, Department of Psychiatry, Massachusetts General Hospital, Boston, MA, United States of America; Division of Adolescent and Young Adult Medicine, Boston Children’s Hospital, Boston, MA, United States of America; Department of Anesthesiology, Perioperative, and Pain Medicine, Mass General Brigham, Harvard Medical School, Boston, MA, United States of America; Department of Anesthesiology, Perioperative, and Pain Medicine, Mass General Brigham, Harvard Medical School, Boston, MA, United States of America; Department of Anesthesiology, Perioperative, and Pain Medicine, Mass General Brigham, Harvard Medical School, Boston, MA, United States of America; Department of Anesthesiology, Perioperative, and Pain Medicine, Mass General Brigham, Harvard Medical School, Boston, MA, United States of America; Department of Anesthesiology, Perioperative, and Pain Medicine, Mass General Brigham, Harvard Medical School, Boston, MA, United States of America; Department of Psychiatry and Behavioral Sciences, Johns Hopkins University School of Medicine, Baltimore, MD, United States of America; Department of Psychiatry and Behavioral Sciences, Johns Hopkins University School of Medicine, Baltimore, MD, United States of America; Department of Psychiatry and Behavioral Sciences, Johns Hopkins University School of Medicine, Baltimore, MD, United States of America; Center for Health Outcomes and Interdisciplinary Research, Department of Psychiatry, Massachusetts General Hospital, Boston, MA, United States of America; Division of Adolescent and Young Adult Medicine, Boston Children’s Hospital, Boston, MA, United States of America; Department of Psychiatry, Harvard Medical School, Boston, MA, United States of America; Department of Anesthesiology, Perioperative, and Pain Medicine, Mass General Brigham, Harvard Medical School, Boston, MA, United States of America

**Keywords:** knee osteoarthritis, childhood abuse, positive childhood experiences, pain, physical functioning

## Abstract

**Objectives –:**

We examined associations of a self-reported history of childhood abuse with pain and physical functioning in patients with knee osteoarthritis (KOA) awaiting total knee arthroplasty (TKA). We also explored the potential moderating effects of positive childhood experiences (PCEs), an index of resilience, on these associations.

**Methods –:**

Prior to TKA, participants with KOA awaiting surgery (*N* = 239) completed self-report measures of adverse childhood experiences (ACEs), PCEs, pain, and physical functioning. We evaluated associations of pain and physical functioning (Brief Pain Inventory [BPI] and Western Ontario and McMaster University of Osteoarthritis Index [WOMAC]) based on the experience of ACEs (childhood abuse), with PCEs (childhood happiness and supportive parental care) as potential moderators.

**Results –:**

Greater exposure to childhood abuse was positively correlated with BPI pain interference as well as WOMAC pain and functioning scores. Additionally, childhood happiness and supportive parental care moderated the positive associations of childhood abuse with pain and physical functioning; though, surprisingly, the adverse effects of childhood abuse on these outcomes were more pronounced among participants with high levels of childhood happiness and supportive parental care.

**Conclusion –:**

Overall, results show an association between a self-reported history of childhood abuse and pain and functioning in patients with KOA awaiting TKA. However, PCEs did not protect against the negative consequences of childhood abuse in our cohort. Further research is needed to validate these associations and gain a more comprehensive understanding of the complex interplay between childhood abuse and PCEs and their potential influences on pain experiences in adults with chronic pain conditions, including KOA.

## 1 Introduction

Knee osteoarthritis (KOA), affecting nearly 365 million individuals worldwide [1,2], is characterized by pain and functional impairment [3]. Identifying factors that contribute to these symptoms is crucial for improving KOA management.

Adverse childhood experiences (ACEs), particularly childhood abuse, are linked to poorer pain and functioning outcomes. Chronic pain sufferers are more likely to report exposures to childhood abuse [4,5], and there is evidence of a prospective link between childhood abuse and the development of chronic pain conditions in adulthood [6,7]. Childhood abuse is also associated with more severe pain symptoms, with numerous studies demonstrating this significant relationship across various populations [8–13]. However, in the context of KOA, the evidence is limited, with only one study exploring this relationship, revealing a connection between childhood abuse and heightened centralized pain (e.g., widespread pain) [12].

Conversely, positive childhood experiences (PCEs) refer to nurturing social interactions and environmental factors encountered during formative years (e.g., supportive parental care) [14]. Importantly, studies have demonstrated a dose-dependent effect of PCEs on various adult health outcomes (e.g., depression) even after accounting for ACEs, indicating a resiliency-promoting role of PCEs [15–18]. Despite this, only a few studies have explored this relationship in the context of chronic pain. In a recent trial, Pugh et al. found that greater exposure to PCEs, particularly those related to social support and networks, was correlated with lower rates of pediatric chronic pain [16]. Furthermore, PCEs moderated the association between ACEs and pain, such that among children exposed to ≤2 ACEs, those who reported ≤5 PCEs were less likely to endorse chronic pain compared to those who reported ≥2 PCEs [16]. In adults with chronic pain, some studies indicate that childhood happiness is associated with higher quality of life and healthier responses to illness, factors linked to better pain outcomes [19,20].

Collectively, these findings align with the Mutual Maintenance Model, which posits that ACEs may create biopsychosocial vulnerabilities that negatively impact physical health, elevating the risk or intensity of chronic conditions like KOA [21]. Conversely, the presence of a chronic condition and its related symptoms can reinforce and aggravate these vulnerabilities, perpetuating the cycle. Importantly, PCEs may serve a protective role, interrupting this cycle of mutual maintenance between the two conditions [22]. This suggests that childhood happiness and supportive parental care could function as safeguards, mitigating the negative consequences of childhood abuse on pain and functioning. However, this has not yet been explored in older adults with chronic pain such as KOA.

This secondary analysis examined associations of a self-reported history of childhood abuse with pain and physical functioning in participants with KOA awaiting total knee arthroplasty (TKA). We hypothesized that greater exposure to childhood abuse would be associated with worse pain and functioning. Exploratory analyses were also conducted to assess the potential moderating effects of childhood happiness and supportive parental care on these associations. We hypothesized that the adverse effects of exposure to childhood abuse on pain and functioning would be attenuated among participants with higher levels of PCEs.

## 2 Methods

### 2.1 Participants

This was a secondary analysis of a parent study assessing biopsychosocial risk factors linked to post-surgical pain after TKA [23]. Participants were recruited from Brigham and Women’s Hospital (BWH, Boston, MA) and Johns Hopkins University (JHU, Baltimore, MD) through various modalities (e.g., flyers) from 2012 to 2018. Inclusion criteria encompassed age ≥45 years, meeting the American College of Rheumatology’s KOA criteria, and proficiency in English. Exclusion criteria included cognitive impairment hindering study completion and a history of specific conditions such as recent myocardial infarction, Raynaud’s disease, severe neuropathy, and severe peripheral vascular disease. A total of 248 participants completed the study. Of these, nine did not complete the questionnaire assessing childhood abuse, thus 239 participants were included in the final analyses.

### 2.2 Measures

Approximately 2 weeks before surgery, participants attended an in-person initial visit and completed self-report questionnaires on demographics, childhood abuse, PCEs, pain, and physical functioning.

#### 2.2.1 Childhood abuse

Exposure to childhood abuse was assessed using a modified version of the standardized ACE questionnaire, which consisted of four items asking about specific types of abuse experienced during the first 18 years of life (i.e., verbal, emotional, physical, and severe physical abuse) on a Likert scale (0 [never] to 4 [very often]) [24]. The total score was averaged (range: 0–4), with higher scores indicating a greater degree of exposure to childhood abuse.

#### 2.2.2 PCEs

A single question, “Overall, how happy was your childhood?” was asked to measure childhood happiness on a Likert scale (0 [not at all happy] to 10 [very happy]) [25]. For supportive parental care, one question was asked, “Overall, how well did your parents take care of you and provide for you during your childhood?” using a scale (0 [not well at all] to 10 [very well]).

#### 2.2.3 Pain and physical functioning

Pain and pain-related interference related to daily activities (e.g., general activity, mood) were assessed using the Brief Pain Inventory (BPI) [26,27], while knee-specific pain and functioning were evaluated using the Western Ontario and McMaster University of Osteoarthritis Index (WOMAC) [28]. The total score for each subscale was averaged (range: 0–10 and 0–100 for the BPI and the WOMAC, respectively), with higher scores indicating worse pain and functioning.

### 2.3 Statistical analyses

Descriptive data are presented as mean values and standard deviations for continuous variables and as percentages for categorical variables (Table 1). Spearman correlations were performed to examine associations of childhood abuse with PCEs and self-reported pain and functioning. To explore the potential protective roles of PCEs on the associations of childhood abuse with pain and physical functioning, moderation analyses were conducted using the PROCESS macros for SPSS [29]. In each model, exposure to childhood abuse was entered as the independent variable, with childhood happiness or supportive parental care as the moderator variable, and pain or physical functioning as the outcome variable. Prior research has shown significant relationships of childhood abuse with gender and race, thus these factors were included as covariates in all moderation analyses [30–32]. All data were analyzed using IBM SPSS Statistics Version 28.

## 3 Results

### 3.1 Participant characteristics

The sample included 239 participants (*M* = 65 years, SD = 8 years; range: 48–87 years). Over half of the participants were female (59.9%) and non-Hispanic White (88.2%). There were no significant associations between childhood abuse and any of the demographic variables. The average score for exposure to childhood abuse was 0.60 (SD = 0.81; range: 0–4), with most participants endorsing generally high levels of childhood happiness (*M* = 7.7, SD = 2.5; range: 0–10) and supportive parental care (*M* = 8.8, SD = 1.9; range: 0–10). Descriptive data for demographics, childhood abuse, PCEs, and pain and functioning outcomes are further depicted in Table 1.

### 3.2 Associations of childhood abuse with PCEs, pain, and physical functioning

Results of spearman correlations showed that greater exposure to childhood abuse was associated with lower childhood happiness (*p* < 0.01) and supportive parental care (*p* < 0.01). Furthermore, BPI Pain Severity, but not Pain Interference scores, were positively correlated with childhood abuse (*p* = 0.03). Both WOMAC Pain (*p* = 0.01) and Physical Functioning scores (*p* = 0.01) were positively associated with childhood abuse. Neither childhood happiness nor supportive parental care were significantly correlated with pain or physical functioning outcomes (*PS* > 0.05; Table 2).

### 3.3 Moderation analyses

Childhood happiness and supportive parental care were assessed as two potential resiliency-promoting factors against exposure to childhood abuse. Specifically, we explored whether these PCEs moderated the associations of childhood abuse with pain and physical functioning while controlling for sex and race.

#### 3.3.1 Childhood happiness

**Figure 1:**
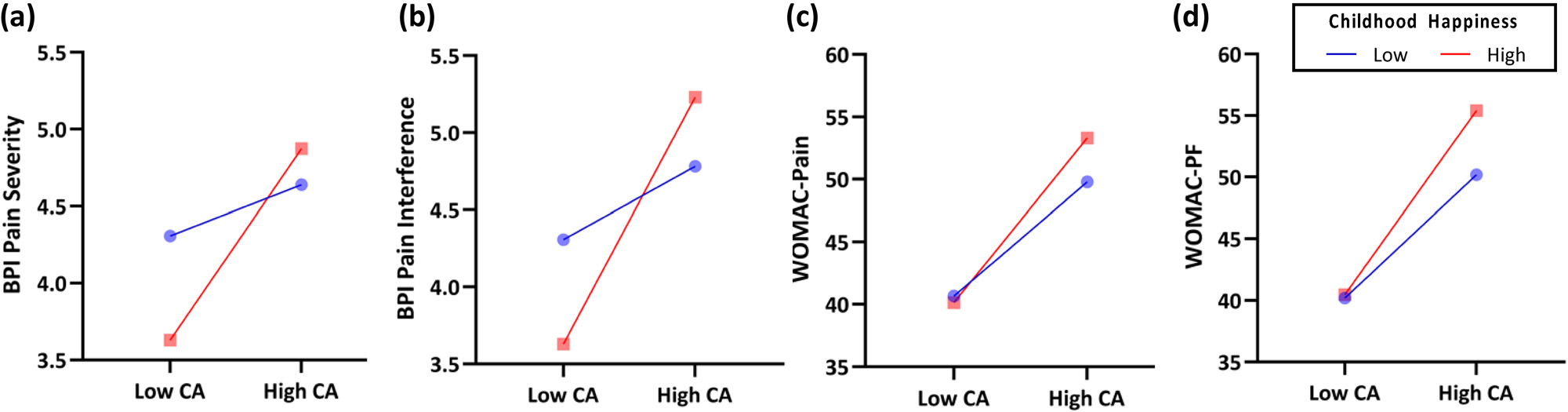
Moderating effects of childhood happiness on the association of childhood abuse with pain and physical functioning, while controlling for sex and race. Childhood happiness as a moderator of (a) the association between childhood abuse and BPI pain severity (higher score = worse pain), (b) the association between childhood abuse and BPI pain interference (higher score = greater interference), (c) the association between childhood abuse and WOMAC pain (higher score = worse pain), and (d) the association between childhood abuse and WOMAC physical functioning (higher score = poorer functioning). CA = childhood abuse; BPI = Brief Pain Inventory; WOMAC = Western Ontario and McMaster University of Osteoarthritis Index; PF = physical functioning.

Childhood happiness significantly moderated the association between childhood abuse and BPI Pain Interference scores (*b* = 0.15, 95% CI [0.02, 0.27], *p* = 0.02), while controlling for sex and race (Figure 1b). Unexpectedly, we found that among participants who endorsed high levels of childhood happiness (i.e., score ≥6.62), greater exposure to childhood abuse was associated with higher BPI Pain Interference scores (*p* ≤ 0.05). In contrast, among those who endorsed low levels of childhood happiness (i.e., score ≤6.50), exposure to childhood abuse and BPI Pain Interference scores were not significantly associated (*p* > 0.05). Childhood happiness did not significantly moderate the associations between exposure to childhood abuse and BPI Pain Severity (*p* = 0.06; Figure 1a), WOMAC Pain (*p* = 0.33; Figure 1c), or WOMAC Physical Functioning scores (*p* = 0.24; Figure 1d).

#### 3.3.2 Supportive parental care

**Figure 2:**
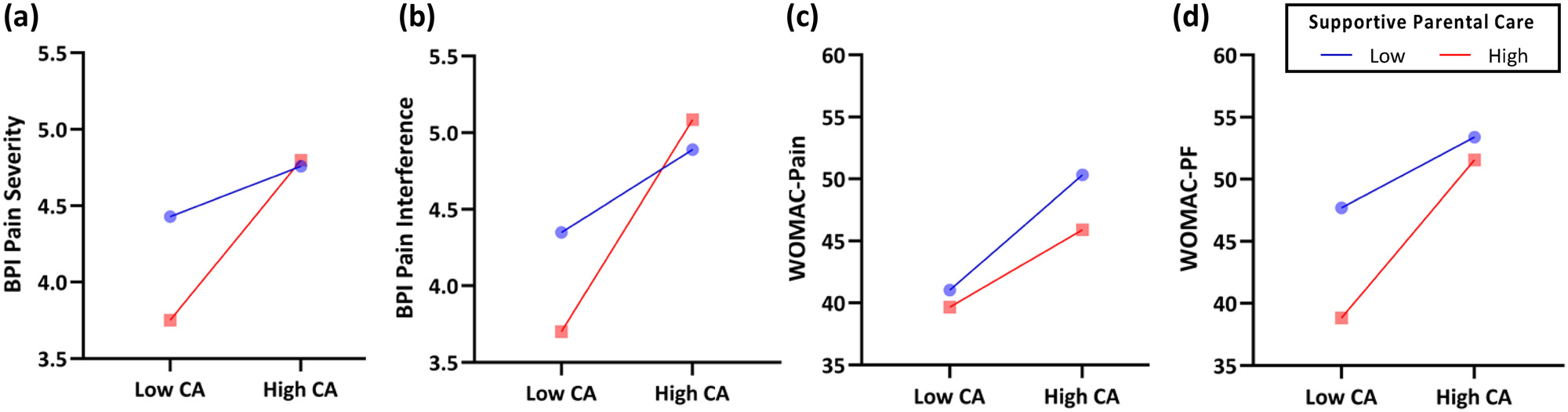
Moderating effects of supportive parental care on the association of childhood abuse with pain and physical functioning, while controlling for sex and race. Childhood happiness as a moderator of (a) the association between childhood abuse and BPI pain severity (higher score = worse pain), (b) the association between childhood abuse and BPI pain interference (higher score = greater interference), (c) the association between childhood abuse and WOMAC pain (higher score = worse pain), and (d) the association between childhood abuse and WOMAC physical functioning (higher score = poorer functioning). CA = childhood abuse; BPI = Brief Pain Inventory; WOMAC = Western Ontario and McMaster University of Osteoarthritis Index; PF = physical functioning.

Supportive parental care significantly moderated the associations of childhood abuse with both BPI Pain Severity (*b* = 0.14, 95% CI [0.02, 0.26], *p* = 0.02; Figure 2a) and BPI Pain Interference scores (*b* = 0.17, 95% CI [0.03, 0.31], *p* = 0.02; Figure 2b), as well as WOMAC Physical Functioning scores (*b* = 1.44, 95% CI [0.24, 2.65], *p* = 0.02; Figure 2d), while controlling for sex and race. Similar to the finding observed with childhood happiness as a moderator, we found that among participants who reported higher levels of supportive parental care during childhood (i.e., score ≥8.69, ≥7.89, ≥7.52, respectively), greater exposure to childhood abuse was associated with higher BPI Pain Severity and Pain Interference as well as worse WOMAC Physical Functioning scores (*PS* ≤ 0.05). Contrastingly, among those who reported low levels of supportive parental care (i.e., score ≤8.50, ≤7.50, ≤7.50, respectively), the associations of childhood abuse with these BPI and WOMAC outcomes were not significant (PS > 0.05). Supportive parental care did not significantly moderate the association between childhood abuse and WOMAC Pain scores (*p* = 0.28; Figure 2c).

## 4 Discussion

In this study, we identified positive correlations between childhood abuse and pain and functioning outcomes in participants with KOA. Our findings align with prior literature that has shown significant links between childhood abuse and more severe pain in individuals with KOA [12] and other chronic pain conditions [10,13,33,34]. For example, Pierce et al. Found significant relationships between childhood abuse and centralized pain, including heightened sensory sensitivity and widespread pain, in participants with KOA [12]. Our results complement and expand upon these by offering preliminary evidence that childhood abuse may also be associated with knee-specific pain in addition to centralized pain. Given that enhanced functioning is a crucial goal in the management of KOA, our result of significant correlations between childhood abuse and functional outcomes further emphasizes the importance of evaluating this relationship in people with KOA. Nonetheless, due to the paucity of existing research on this topic, further investigation is needed.

Previous research has shown that PCEs can serve as a resiliency-promoting factor, mitigating negative effects of ACEs on health outcomes among adults [15–17,35]. A recent study focused on pediatric chronic pain also yielded similar results [16]. In contrast, we found that the adverse effects of childhood abuse on pain and physical functioning were more pronounced among participants with high levels of childhood happiness and supportive parental care. One possible explanation for this discrepancy is the differences in the study design. Specifically, Pugh et al. examined the cumulative effects of neglect and family dysfunction [16,36] whereas we assessed those of childhood abuse only. Consequently, the inconsistent findings may suggest that different ACE categories may have varying effects on pain outcomes in the context of PCEs. However, due to the lack of research in this area, it is challenging to ascertain the extent to which these divergent effects may have contributed to observed inconsistencies. Thus, additional research is needed to examine these relationships more closely.

Nevertheless, our preliminary findings highlight the complexity of the relationships between childhood abuse and PCEs and suggest considering the broader context in which childhood abuse occurs may be important when working with patients with pain. For instance, in the present study, it may seem paradoxical that participants who reported high levels of childhood abuse also reported higher degrees of PCEs. Yet, a more nuanced evaluation of this finding by considering contextual factors may offer clarity. For example, in two-parent households, an individual may be recalling abuse by one parent while receiving support from another. Alternatively, an abusive parent may intermittently display loving and supportive behavior while remaining emotionally or physically abusive. Noteworthy is the latter scenario, as evidence suggests that liability in the parent’s behaviors, even when accompanied with positive parenting practices, may be damaging especially among those exposed to childhood abuse [37]. Thus, future research that employs assessments capturing the nuanced details of these experiences is necessary to fully understand the relationships between childhood abuse, PCEs, and pain outcomes in adulthood.

Several important limitations should be acknowledged. Given the cross-sectional nature of the present study, we are unable to establish causality. Although we recruited participants from two large academic medical centers in distinct geographic regions, our sample was largely homogenous (i.e., White female). Given the well-documented racial and ethnic disparities in childhood abuse and KOA [32], future studies should investigate these relationships using a more diverse and encompassing sample. Moreover, our study focused on older adults aged 45 and above with a specific chronic pain condition, limiting generalizability of our findings to younger populations or those with other types of chronic pain.

Lastly, we acknowledge that the self-report measure used to assess the degree of exposure to childhood abuse in our study may not have been sensitive enough to fully capture the nuanced understanding of how childhood abuse is related to pain, physical functioning, and PCEs. Additionally, definitions and norms related to childhood abuse have shifted over time, which may further impact self-reporting prevalence and the most accurate reports of childhood abuse [38]. Future longitudinal studies and those employing mixed methodology are needed to better understand how older adults conceptualize childhood abuse. Such methods would allow for a more nuanced understanding of the ages, frequency, and situations around which exposure to childhood abuse occurred, allowing for a more comprehensive understanding of how these experiences influence the pain experience.

## 5 Implications

Given the relationship between exposure to childhood abuse and pain, clinical providers should consider adopting trauma-informed and trauma-focused approaches. These include screening for a history of childhood abuse in patients presenting with painful conditions like KOA and referring those with such a history to appropriate psychosocial interventions such as cognitive behavioral therapy (CBT) and acceptance and commitment therapy (ACT) [39]. CBT aims to modify negative thought patterns and behaviors [40]. Meanwhile, ACT encourages an open, non-judgmental acceptance of experiences, emphasizing psychological flexibility, mindfulness, and actions aligned with personal values [41]. Evidence supports the efficacy of these interventions in reducing pain, enhancing functionality, and improving well-being across various chronic pain conditions [42,43]. Notably, these interventions target common psychological underpinnings resulting from childhood abuse and pain, such as negative affect and catastrophizing, making them valuable tools in pain management for individuals with a history of childhood abuse [43,44]. Furthermore, incorporating trauma-specific therapies like prolonged exposure (PE) and cognitive processing therapy (CPT) can offer additional benefits. PE helps patients gradually confront trauma-related memories [45], while CPT assists in the cognitive restructuring of trauma-related thoughts [46]. Integrating these interventions with standard pain management offers a comprehensive, multimodal strategy that effectively addresses the complex relationship between KOA and childhood abuse, significantly enhancing pain management outcomes.

## 6 Conclusion

We found that a self-reported history of childhood abuse was associated with worse pain and poorer physical functioning among older adults with KOA, and that the relationships between childhood abuse and worse outcomes were strongest among those with high levels of childhood happiness and parental care. These results suggest that childhood abuse and PCEs may impact the pain experience in adulthood and highlight important opportunities to optimize the treatment for KOA.

## Figures and Tables

**Table 1:** Patient characteristics

	Full sample (*N* = 239)

Age	65.0 ± 8.2
**Gender**	
Female	59.4%
**Race**	
White	88.2%
Black	8.9%
Asian	1.7%
American Indian/Alaskan Native	0.4%
More than one race	0.8%
**Ethnicity**	
Hispanic/Latino	99.6%
Non-Hispanic	0.4%
Childhood abuse	0.6 ± 0.8
**PCEs**	
Childhood happiness	7.7 ± 2.5
Supportive parental care	8.8 ± 1.9
**BPI**	
Pain severity	4.3 ± 2.2
Pain interference	4.3 ± 2.5
**WOMAC**	
Pain	45.3 ± 22.6
Physical functioning	46.3 ± 23.2

PCEs = positive childhood experiences; BPI = Brief Pain Inventory; WOMAC = Western Ontario and McMaster University of Osteoarthritis Index.

**Table 2:** Correlations of childhood abuse with PCEs, pain, and physical functioning

	Childhood abuse	Childhood happiness	Supportive parental care	BPI severity	BPI interference	WOMAC-pain	WOMAC-PF

Childhood abuse	—						
Childhood	−0.54**	—					
happiness Supportive parental care	−0.46**	0.71**	—				
BPI severity	0.12	−0.04	−0.05	—			
BPI interference	0.15*	−0.03	−0.03	0.68**	—		
WOMAC-pain	0.16*	0.00	−0.05	0.73**	0.71**	—	
WOMAC-PF	0.17**	0.02	−0.11	0.70**	0.68**	0.81**	—

* *p* < 0.05

** *p* < 0.01

PCEs = positive childhood experiences, BPI = Brief Pain Inventory; WOMAC = Western Ontario and McMaster University of Osteoarthritis Index; PF = physical functioning.

## Data Availability

The raw data can be obtained on request from the corresponding author.
